# Circadian clock is associated with tumor microenvironment in kidney renal clear cell carcinoma

**DOI:** 10.18632/aging.103509

**Published:** 2020-07-18

**Authors:** Liangcheng Zhou, Zhili Luo, Zuwei li, Qinying Huang

**Affiliations:** 1 Department of Nephrology, Maoming People’s Hospital, Maoming 525000, China; 2 Department of Rehabilitative Medicine, Gaozhou People’s Hospital, Maoming 525200, China; 3 Department of Urology, Gaozhou People’s Hospital, Maoming 525200, China; 4 Department of Ophthalmology, Shantou University Medical College, Shantou 515041, China

**Keywords:** circadian clock, KIRC, immunotherapy, tumor microenvironment, biomarker

## Abstract

Background: Kidney renal clear cell carcinoma (KIRC) is one of the most prevalent malignancies with high incidence and mortality. The circadian clock, which is also involved in the regulation of the immune system and tumor microenvironment, is an internal timing system that allows organisms to adjust biological processes and behaviors according to geophysical time.

Result: A wide range of circadian clock genes are epigenetically altered in KIRC, and associated with the overall survival and disease-free survival of patients. SNV analysis revealed missense mutation and splice site to be the most common variant types of circadian clock genes in KIRC. Several circadian clock genes were involved in the regulation of some cancer-related hallmark pathways, including apoptosis and cell cycle pathway. Further, immune infiltrates analysis not only revealed that the expression of circadian clock genes is associated with immune cell infiltrates, but also that somatic copy-number alteration of circadian clock genes could inhibit the immune infiltrates. Moreover, enrichment analysis implied that the circadian clock genes could regulate transcription factor activity and circadian rhythm in KIRC.

Conclusion: Our results demonstrate the potential of chrono-immunotherapy as a candidate option for the management of KIRC.

Method: Multi-omics analysis was performed to comprehensively determine the roles of core circadian clock genes in KIRC.

## INTRODUCTION

Kidney cancer is one of the most prevalent malignancies, with over 400,000 new cases, and 175,000 cancer-related deaths occurring annually across the globe [1]. Kidney renal clear cell carcinoma (KIRC) the most common subtype of kidney cancers, accounting for >70% of all kidney cancers. The incidence of KIRC is on the rise, and about 20% of patients exhibit distant metastases at the initial diagnosis [2]. Despite the application of various therapy approaches, the prognosis of KIRC remains unsatisfactory, with median overall survival of 10–15 months in stage IV patients [3]. Therefore, there is a critical need for innovative approaches for the diagnosis, therapy, and prognosis of KIRC.

The circadian clock is an internal timing system that enables organisms to adjust biological processes and behaviors according to geophysical time [4]. Genes involved in the circadian clock system play a vital role in biological processes including DNA damage and repair, cell proliferation, and metastasis, thus affecting tumorigenesis and progression [5]. Besides, accumulating evidence has unraveled the significance of the circadian clock genes in the diagnosis, therapy, and prognosis of cancers. For instance, circadian clock gene BMAL1 suppresses tumorigenesis and increases paclitaxel sensitivity in tongue squamous cell carcinoma [6]. Another study revealed that circadian clock gene Per2 is a potential biomarker for the prognosis of gastric cancer [7]. However, in primary KIRC, little is known about the role of circadian clock genes.

Accumulating evidence has demonstrated that the circadian clock is involved in the regulation of the immune system and tumor microenvironment [8]. A previous study reported the adaptive immune response to immunization and pathogens to be time-dependent [9]. Another study found that the circadian clock gene CLOCK promotes tumor cell metabolism and microglia infiltration into the tumor microenvironment, flagging CLOCK as a novel therapeutic target for Glioblastoma [10]. The relationship between the circadian clock and tumor microenvironment has also been demonstrated in thoracic cancers [8]. We, therefore, sought to analyze the role of the circadian clock in KIRC tumor microenvironment.

In current study, the expression and clinical significance of circadian clock genes such as CLOCK, BMAL1 (ARNTL), CRY1, CRY2, NR1D1, PER1, PER2, PER3, and RORA, were explored using various online bioinformatics tools. Further, we comprehensively analyzed the variation, drug resistance, immune infiltration, and functional enrichment of circadian clock genes in the primary KIRC tumor microenvironment. Our findings provide more insight on the crosstalk between tumor microenvironment and the circadian clock in primary KIRC.

## RESULTS

### Defining core circadian clock genes in KIRC and normal tissue

**Figure 1:**
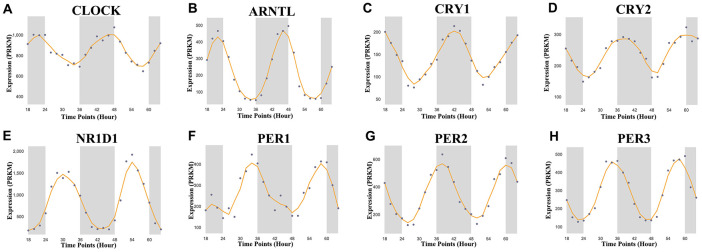
**Core circadian clock genes in KIRC.** The circadian rhythm of core circadian genes in KIRC, including CRY2, PER1, NR1D1, CLOCK, CRY1, PER3, PER2, and ARNTL. There is no enough data of RORA in KIRC. KIRC, Clear cell renal cell carcinoma.

**Figure 2:**
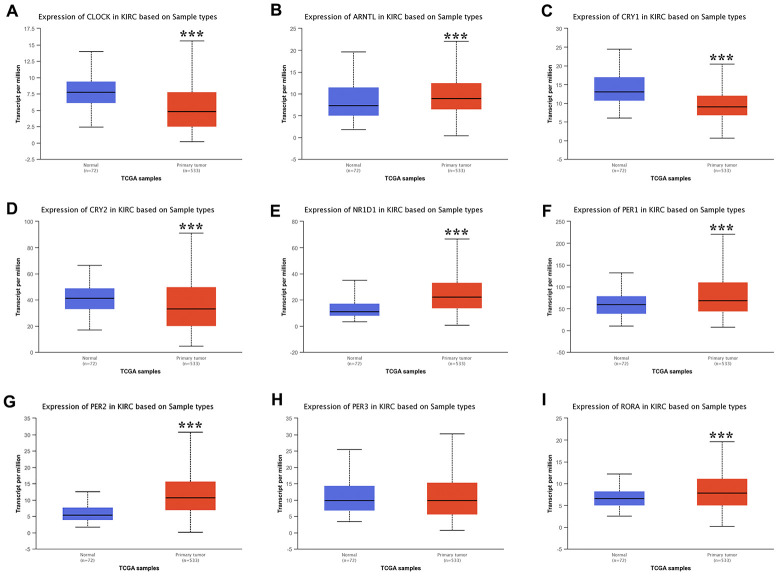
**The expression of core circadian clock genes in KIRC tissue and normal tissue.** ***p<0.001. KIRC, Clear cell renal cell carcinoma.

**Figure 3:**
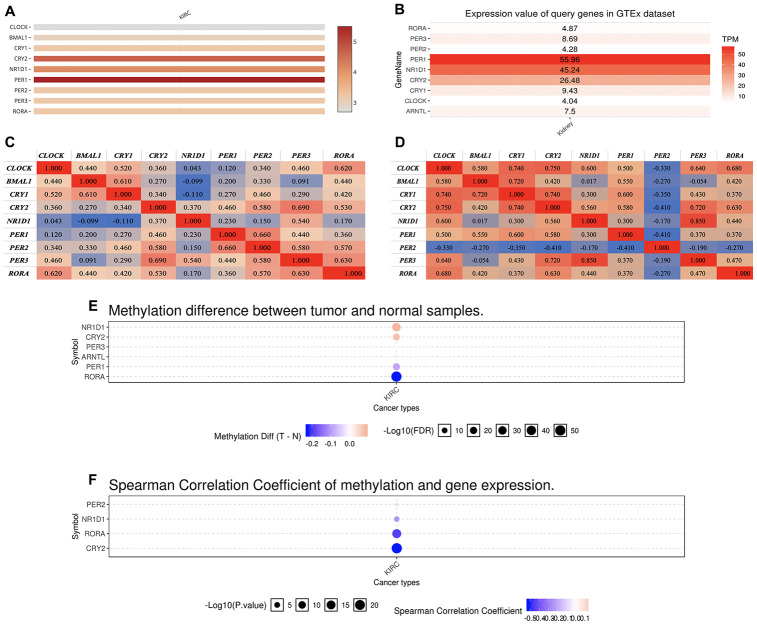
**The expression of core circadian clock genes in KIRC tissue and normal tissue.** (**A**) relative expression of circadian clock genes in tumor tissues. (**B**) relative expression of circadian clock genes in normal tissues. (**C**) Co-expression of circadian clock genes in tumor tissues. (**D**) Co-expression of circadian clock genes in normal tissues. (**E**) The methylation difference between tumors and normal tissues. (**F**) The methylation level affects the core circadian gene expressions. KIRC, Clear cell renal cell carcinoma.

In this study, we selected a total of nine previously described core circadian clock genes, namely; CLOCK, BMAL1, CRY1, CRY2, NR1D1, PER1, PER2, PER3, and RORA [11, 12]. By exploring the expression profile of the core circadian genes in kidney tissue at different intervals using RNA-seq, [13] we observed fluctuation in their expression (Figure 1). However, there was not enough data about the expression profile of RORA. Exploring the mRNA level of these genes in KIRC revealed a significant upregulation of ARNTL, NR1D1, PER1, PER2, and RORA compared with normal tissues (Figure 2, p<0.001). Moreover, the mRNA expression of CLOCK, CRY1, and CRY2 declined in tumor tissues compared with normal tissues (Figure 2, p < 0.001). Further, relative expression of circadian clock genes in KIRC tissues and kidney tissues showed PER1 and CLOCK genes as the highest and lowest expressed, respectively, in KIRC tissues (Figure 3A). Besides, based on the GETx dataset, expression of PER1 and RORA genes respectively lead and trail in kidney tissues (Figure 3B). In KIRC tissues, co-expression analysis revealed a low to high positive correlation among expression of the circadian clock genes, except for NR1D1 (Figure 3C). However, in normal kidney tissues, co-expression analysis demonstrated a moderate to high positive correlation among the expression of these circadian clock genes, except for PER2 (Figure 3D). The difference in methylation levels between KIRC and normal tissues was also detected, with the methylation levels of NR1D1, CRY2, PER1, and RORA exhibiting significant difference (Figure 3E). Figure 3F shows the effect of methylation on the downregulation of NR1D1, RORA, and CRY2 in KIRC. Collectively, the above results implied epigenetic alteration of core circadian clock genes in KIRC.

### The prognostic value of circadian clock genes in KIRC

**Figure 4:**
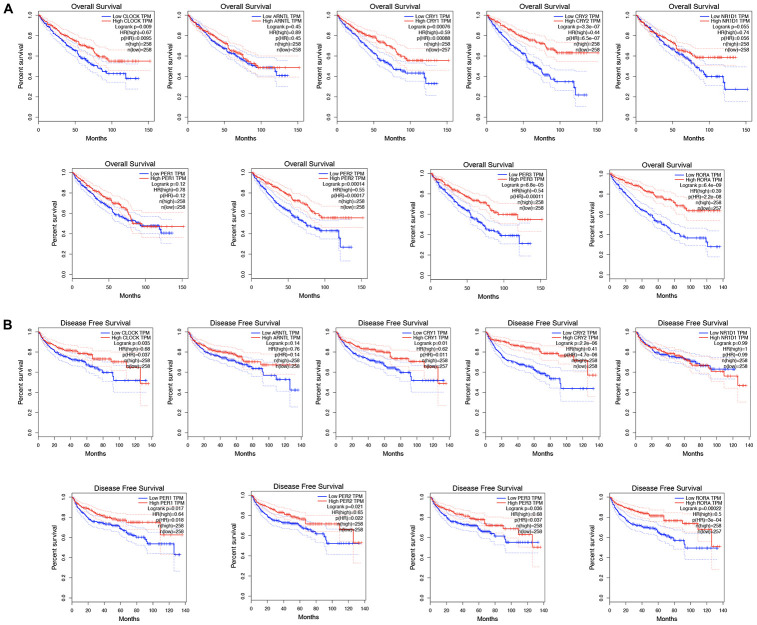
**The prognostic value of circadian clock genes in KIRC.** (**A**) The role of circadian clock genes in the overall survival of KIRC patients. (**B**) The role of circadian clock genes in the disease-free survival of KIRC patients. KIRC, Clear cell renal cell carcinoma.

Besides affecting cancer development and having a therapeutic effect, increasing evidence shows that circadian genes are significantly associated with prognosis [14, 15]. We conducted a comprehensive evaluation of circadian clock genes in the prognosis in KIRC, and as a result, KIRC patients who highly expressed CLOCK (HR=0.67, p = 0.009), CRY1 (HR=0.59, p = 0.00076), and CRY2 (HR=0.44, p = 3.3e-07) had a better overall survival (Figure 4A). Similarly, KIRC patients with highly expressing PER2 (HR=0.55, p = 0.00014), PER3 (HR=0.54, p = 8.8e-05), and RORA (HR=0.39, p = 6.4e-09) had a better overall survival (Figure 4A). In disease free survival analysis, KIRC highly expressing CLOCK (HR=0.68, p = 0.035), CRY1 (HR=0.62, p = 0.01), and CRY2 (HR=0.41, p = 2,2e-06) had a better disease-free survival (Figure 4B). Similarly, KIRC patients who highly expressed PER1 (HR=0.64, p = 0.017), PER2 (HR=0.65, p = 0.021), PER3 (HR=0.68, p = 0.036), and RORA (HR=0.5, p = 0.00022) recorded a better disease-free survival (Figure 4B).

### The role of the circadian clock in the cancer hallmarks in KIRC

**Figure 5:**
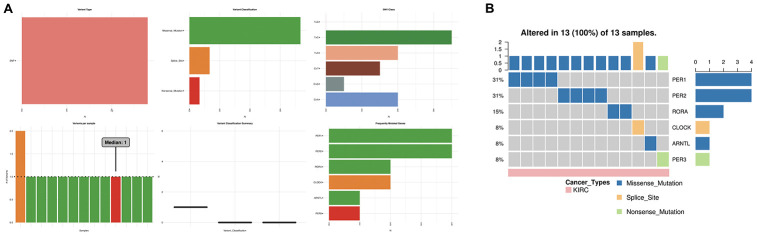
**The SNV analysis of circadian clock genes in KIRC.** (**A**) summary plot displays SNV frequency and variant types of circadian clock genes in KIRC. (**B**) waterfall plot shows the mutation distribution of circadian clock genes in KIRC and a SNV classification of SNV types. KIRC, Clear cell renal cell carcinoma; SNV, single nucleotide variation.

**Figure 6:**
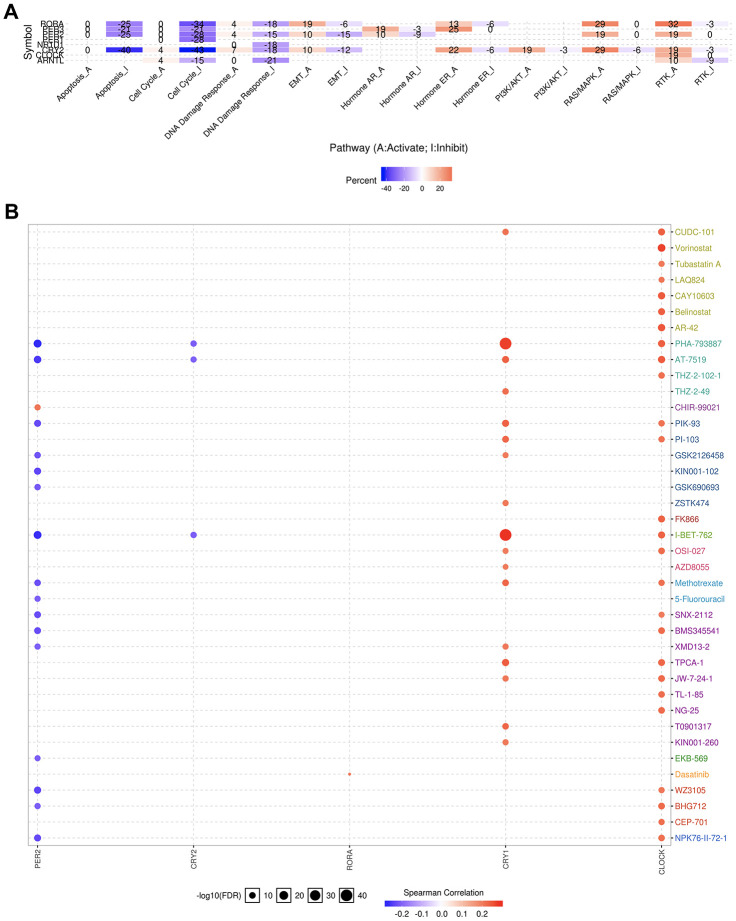
**The cancer related pathways and drug resistance analysis of circadian clock genes in KIRC.** (**A**) the role of circadian clock genes in the famous cancer related pathways. (**B**) The Spearman correlation represent the gene expression correlates with the drug. The positive correlation means that the gene high expression is resistant to the drug, vise verse. KIRC, Clear cell renal cell carcinoma.

We further evaluated the role of the circadian clock in the cancer hallmarks in KIRC to reveal the potential effects of circadian disruption in KIRC. SNV analysis revealed missense mutation and splice site to be the most common variant types of circadian clock genes in KIRC (Figure 5). Additionally, we examined the role of circadian clock genes in cancer-related hallmark pathways such as TSC/mTOR, RTK, RAS/MAPK, PI3K/AKT, Hormone ER, Hormone AR, EMT, DNA Damage Response, Cell Cycle, and Apoptosis pathways. Our findings demonstrated that circadian clock genes were closely related to most of these cancer-related hallmark pathways in KIRC (Figure 6A). Specifically, circadian clock genes were involved in the suppression of apoptosis, cell cycle, and DNA damage response pathways (Figure 6A). And circadian clock genes were involved in the activation of the Hormone ER pathway, RAS/MAPK pathway, and RTK pathway (Figure 6A).

An explicit relationship between clock genes and existing drug targets is the precondition and basis of cancer chronotherapy. Besides, a clock-related drug administration following a circadian timing, may significantly improve the efficacy and reduce toxicity. From our correlation analysis between circadian clock genes expression and drug sensitivity, we found high expression of CLOCK and CRY1 to be associated with drug resistance (Figure 6B). However, low expression of PER2 was CRY2 is associated with drug resistance (Figure 6B).

### Immune infiltrates analysis of circadian clock in KIRC

**Figure 7:**
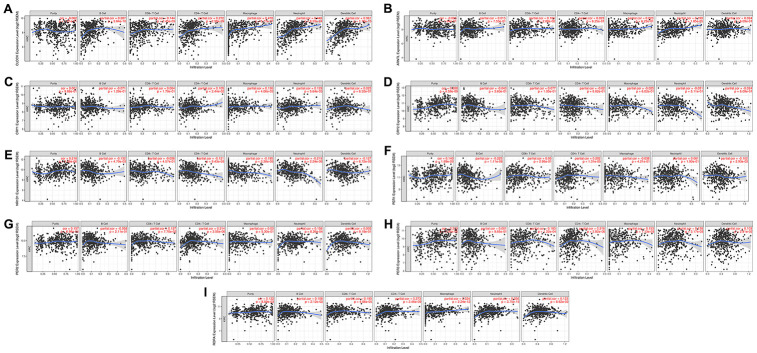
**The correlation between circadian clock genes and the immune infiltration level in KIRC.** KIRC, Clear cell renal cell carcinoma.

**Figure 8:**
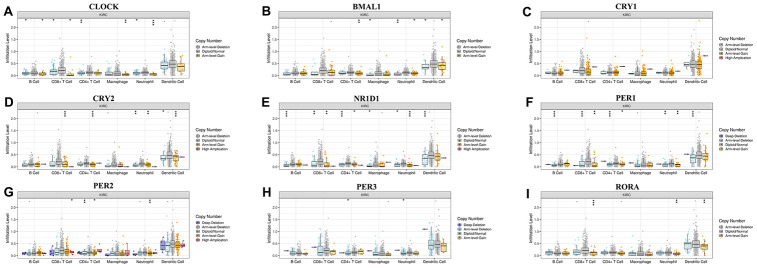
**Associations of circadian clock genes alteration with immune cells infiltration.**

The emergence of immunotherapy using drugs targeting immune checkpoints has generated tremendous promise for KIRC patients, especially for patients in advanced stages [16]. Although a previous study revealed the circadian rhythm of several immune checkpoints in thoracic cancer [8], the role of the circadian clock in immune checkpoints and immunotherapy remains unclear. In the current study, our analysis of the correlation between the abundance of six immune cell types, and circadian clock expression revealed the expression of CLOCK to be positively associated with the infiltration abundance of B cells (r=0.287, P=3.84e-10), CD8+ T cells (Cor=0.144, P=2.45e-03), CD4+ T cells (r=0.272, P=2.98e-09), macrophages (r=0.478, P=5.77e-27), Neutrophils (r=0.446, P=8.29e-24), and Dendritic cells (r=0.361, P=1.76e-15) (Figure 7A). Expression of ARNTL positively correlated with the abundance of CD8+ T cells (r=0.106, P=2.68e-02), and Neutrophils (r=0.199, P=1.85e-05) as shown in Figure 7B. Moreover, CRY1 expression was positively correlated with three immune cell infiltrates including CD4+ T cells, macrophages, and Neutrophils (Figure 7C). However, there was no significant correlation between CRY2 expression and the six immune cells' abundance (Figure 7D). Interestingly, the abundance of five immune cells namely; B cells (r= -0.132), CD4+ T cells (r=-0.121), macrophages (r=-0.195), Neutrophils (r=-0.219), and Dendritic cells (r=-0.127)) was negatively associated with NR1D1 expression (Figure 7E). Correlation between PER1 expression with the abundance of B cells and CD4+ T cells, were respectively significant at (r= -0.225, P=1.11e-06) and (r=0.202, P=1.31e-05) (Figure 7F). CD8+ T cells, CD4+ T cells, Neutrophils were correlated with PER2 expression (Figure 7G). In KIRC, PER3 (Figure 7H) and RORA (Figure 7I) expression showed a significant correlation with CD8+ T cells, CD4+ T cells, macrophages, Neutrophils, and Dendritic cells. Moreover, we observed that except for CRY1, somatic copy-number alteration (SCNA) of circadian clock genes could commonly inhibit the immune infiltrates such as CD8+ T cell, neutrophil cell, dendritic cell, macrophage, CD4+ T cell, and B cell (Figure 8). These results indicated a possible association between the circadian clock and immune infiltrates in KIRC.

### Enrichment analysis of circadian clock in KIRC

**Figure 9:**
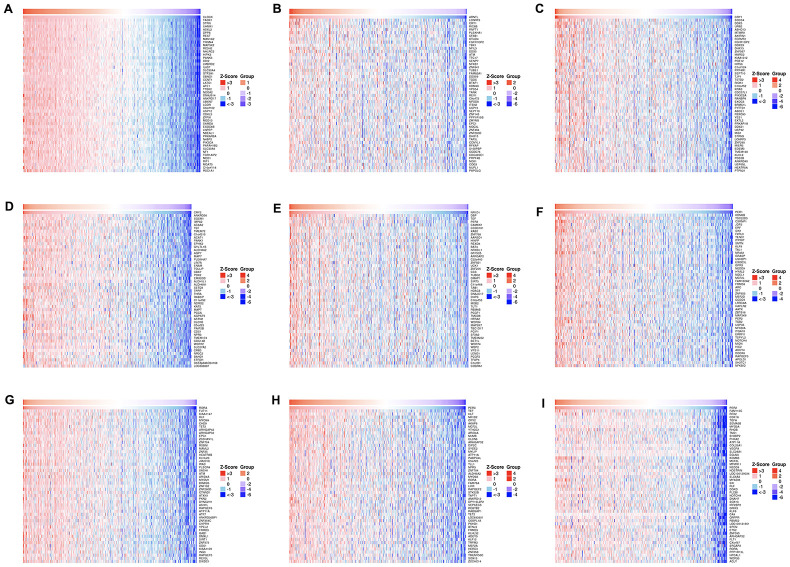
**The top 50 genes most associated with circadian clock genes.**

**Figure 10:**
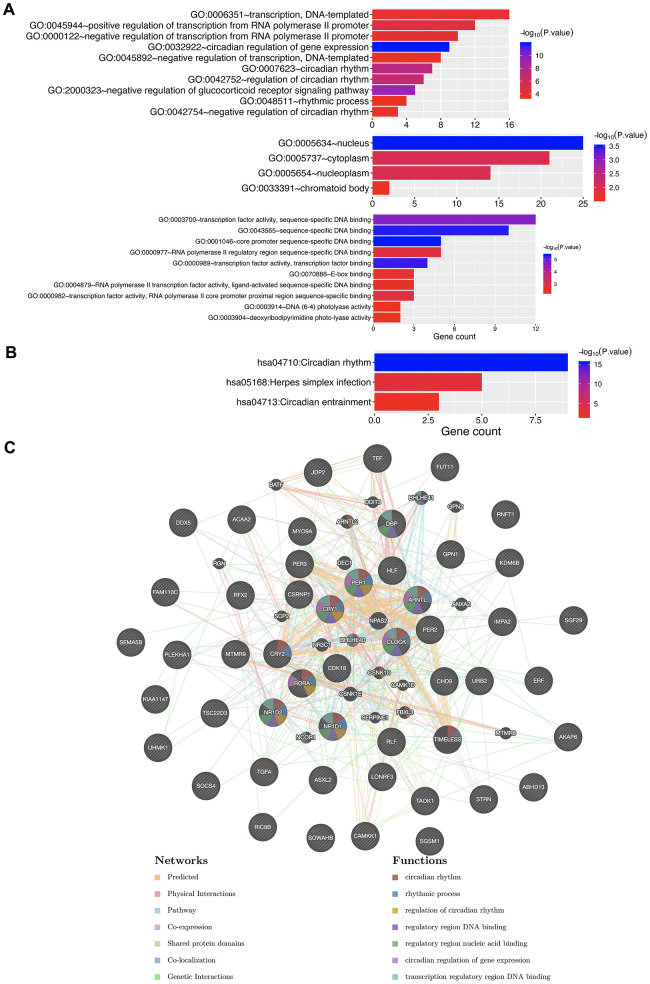
**The enrichment analysis of circadian clock genes and neighboring genes in KIRC.** (**A**) GO enriched terms. (**B**) KEGG enriched terms. (**C**) PPI network. KIRC, Clear cell renal cell carcinoma; GO, Gene Ontology; KEGG: Kyoto Encyclopedia of Genes and Genomes; BP: Biological processes; CC: Cellular component; MF: Molecular functions; PPI, Protein-protein interaction.

Due to the significance of circadian clock genes in KIRC, we explored their potential functions in KIRC. The top ten genes most closely associated with circadian clock genes were extracted, and are outlined in Figure 9 and Table 1. Upon submission of the circadian clock genes and the ten associated genes to DAVID for GO and KEGG analysis, we observed that circadian clock genes were involved in transcription, circadian rhythm, nucleus, and transcription factor activity in GO function analysis (Figure 10A). KEGG pathways analysis revealed circadian clock genes to be responsible for circadian rhythm, herpes simplex infection, and circadian entrainment (Figure 10B). Moreover, the result of PPI network suggested that circadian clock genes are associated with circadian rhythm, rhythmic process, regulatory region DNA binding, and nucleic acid binding (Figure 10C). These results implied the involvement of circadian clock genes in regulating transcription factor activity and circadian rhythm in KIRC.

**Table 1:** The top 5 significant gene correlated with circadian clock in KIRC.

**circadian clock**	**Correlated genes**
CLOCK	TOAK1, STRN, UHMK1, ASXL2, DPP8
ARNTL	LONRF3, CRY1, RIC8B, RNFT1, PLEKHA1
CRY1	SOCS4, DDX5, URB2, ABHD13, MTMR9
CRY2	ANKRD56, SGSM1, IMPA2, ACAA2, TEF
NR1D1	DBP, TEF, PER3, CAMKK1, CCDC101, XAB1
PER1	KDM6B, TSC22D3, CSRNP1, JDP2, ERF
PER2	FAM110C, RFX2, CDK18, TGFA, SEMA5B
PER3	TEF, HLF, NR1D2, CRY2, AKAP6
RORA	FUT11, KIAA1147, RLF, MYO9A, CHD9

## DISCUSSION

Disruption of biorhythms can lead to physiological disorders of homeostasis in mammals, which are often associated with several pathologies, including cancer [17]. Besides showing signaling networks that affect cancer pathogenesis and progression, previous studies have described the connection between circadian clock disruption and various biological processes [18, 19]. Circadian rhythm control is thought to be easily affected by tumor microenvironment metabolism and systemic metabolism [20], hence we applied multi-omics tools in evaluating the role of the circadian clock in primary KIRC.

Since the circadian clock is operated by genes, and circadian clock expression largely affects the function of the circadian clock [14], we first detected the circadian clock genes expressed in KIRC. The findings revealed that a high fraction of clock molecules were dysregulated in KIRC tissues, indicating that circadian rhythms were disrupted and the tumor microenvironment altered to favor the pathogenesis and progression of KIRC. Moreover, the dysregulation of a high fraction of clock molecules was associated with the prognosis of KIRC patients. Therefore, clarifying the circadian clock state in the KIRC microenvironment is essential in establishing the specific times during circadian rhythms that KIRC patients could be treated.

Another important finding of our study was that dysregulation of the circadian clock is related to several hallmark cancer-related pathways. We found that circadian clock genes were associated with suppression of the cell cycle pathway, apoptosis pathway, and DNA damage response pathway in KIRC. Enrichment analysis further suggested that circadian clock genes were involved in the regulation of DNA binding, and gene expression in KIRC. Besides, a previous study suggested that the clock system imbalance could mediate cancer susceptibility through several biological behaviors, including DNA damage, repair mechanisms, and apoptosis [21]. Although the cell cycle and circadian clock are considered as two different biological oscillators, their close relation and interaction have been reported [22].

Circadian variations of various aspects of the immune system have been previously described, except for the role of the circadian clock in immune cells. In our study, we highlight the strong correlation between the circadian clock and immune cells, including B cell, CD4 T cell, CD8 T cell, neutrophil, macrophage, and dendritic cell. In line with Thomas’s study, CD4+ T cell responses could be mediated by circadian rhythm, and the intrinsic cellular circadian oscillator could drive rhythmic CD4+ T cell immune responses [23]. In macrophages, the immune checkpoint pathway is regulated by the circadian clock [24].

Our study had the following limitations. First, our analysis focused on the expression of clock genes at the transcriptional level and their clinical significance, yet transcriptomics analysis can only reflect some aspects, rather than the global alterations. Second, the expression profile of RORA in the kidney is still unclear, and lastly, the data of different patients may have been collected at different times, which could affect our findings.

## CONCLUSION

In conclusion, a high fraction of clock molecules were dysregulated in KIRC tissues and associated with the prognosis of patients. A significant association was revealed between circadian clock genes and cancer hallmark pathways, as well as immune infiltrates. Our findings provide additional data on the application of immune checkpoint inhibitors based on circadian timing.

## MATERIALS AND METHODS

### Data sets and data availability

KIRC samples were extracted from The Cancer Genome Atlas (TCGA: https://cancergenome.nih.gov), and the clinical information from the UCSC Xena (https://xenabrowser.net/hub/). Data of the Genotype-Tissue Expression (GTEx: https://gtexportal.org) were used to represent expression in the normal renal tissues. The mRNA expression, methylation, and single nucleotide variation were analyzed in KIRC samples (n=533) with complete corresponding clinical information.

### Gene expression, SNV, Methylation, pathway activity, and drug sensitivity analysis

The significance of expression analysis was analyzed using a |Fold Change| > 1 and a p-value < 0.05. Maftools was performed to generate Single Nucleotide Variation (SNV) summary and oncoplot waterfall plot of circadian clock genes [25]. Student’s T-test was performed to determine the methylation difference between tumor and normal samples, with significance set at FDR ≤ 0.05. A total of 265 small molecules or drugs were obtained from the Genomics of Drug Sensitivity in Cancer (GDSC: https://www.cancerrxgene.org/), and the Pearson correlation coefficient was performed to analyze the correlation between expression and drug sensitivity. In pathway activity analysis, gene expression was divided into high and low expression, and if the PAS of Gene A high group was larger than the PAS of Gene A low group, gene A was considered to have an activation impact on that pathway [26].

### Prognosis analysis

The significance of each circadian clock gene in the overall survival (OS) and disease-free survival (RFS) was analyzed using Kaplan-Meier curves, and a median value of gene expression was set as the group cutoff separating the high expression and low expression groups. Hazards ratio, 95% Confidence Interval, and log-rank P were also analyzed using the Cox PH model.

### Immune infiltrate analysis

The significant presence of six immune cell types namely; B cell, CD4 T cell, CD8 T cell, neutrophil, macrophage, and dendritic cell, in the tumor microenvironment has been previously reported [26]. Therefore, we applied CIBERSORT, which is an established algorithm, to estimate the correlation between abundance, and gene expression [27].

### Enrichment analysis

Using Pearson correlation test results based on the TCGA KIRC sample, we extracted the top ten most significant genes that were positively associated with circadian clock genes. Thereafter, circadian clock genes and the ten selected genes were submitted to DAVID 6.8 (https://david.ncifcrf.gov/) for enrichment analysis, including Gene Ontology (GO) and Kyoto Encyclopedia of Genes and Genomes (KEGG) pathway. The results were processed and visualized in the R project using the “ggplot2” package, and, a P < 0.05.
